# Characterization of cognitive function in survivors of diffuse gliomas using resting-state functional MRI (rs-fMRI)

**DOI:** 10.1007/s11682-021-00497-6

**Published:** 2021-08-05

**Authors:** Chencai Wang, Kathleen Van Dyk, Nicholas Cho, Catalina Raymond, Justin Choi, Noriko Salamon, Whitney B. Pope, Albert Lai, Timothy F. Cloughesy, Phioanh L. Nghiemphu, Benjamin M. Ellingson

**Affiliations:** 1 UCLA Brain Tumor Imaging Laboratory (BTIL), Center for Computer Vision and Imaging Biomarkers, David Geffen School of Medicine, University of California Los Angeles, 924 Westwood Blvd., Suite 615, Los Angeles, CA 90024 USA; 2 Department of Radiological Sciences, David Geffen School of Medicine, University of California Los Angeles, Los Angeles, CA USA; 3 Medical Scientist Training Program, David Geffen School of Medicine, University of California Los Angeles, Los Angeles, CA USA; 4 Department of Psychiatry and Biobehavioral Sciences, David Geffen School of Medicine, Semel Institute, University of California Los Angeles, Los Angeles, CA USA; 5 Department of Neurology, David Geffen School of Medicine, University of California Los Angeles, Los Angeles, CA USA

**Keywords:** Diffuse gliomas, Cognitive function, Resting-state fMRI, Connectivity, Daily functioning

## Abstract

As treatments for diffuse gliomas have advanced, survival for patients with gliomas has also increased. However, there remains limited knowledge on the relationships between brain connectivity and the lasting changes to cognitive function that glioma survivors often experience long after completing treatment. This resting-state functional magnetic resonance imaging (rs-fMRI) study explored functional connectivity (FC) alterations associated with cognitive function in survivors of gliomas. In this pilot study, 22 patients (mean age 43.8 ± 11.9) with diffuse gliomas who completed treatment within the past 10 years were evaluated using rs-fMRI and neuropsychological measures. Novel rs-fMRI analysis methods were used to account for missing brain in the resection cavity. FC relationships were assessed between cognitively impaired and non-impaired glioma patients, along with self-reported cognitive impairment, non-work daily functioning, and time with surgery. In the cognitively non-impaired patients, FC was stronger in the medial prefrontal cortex, rostral prefrontal cortex, and intraparietal sulcus compared to the impaired survivors. When examining non-work daily functioning, a positive correlation with FC was observed between the accumbens and the intracalcarine cortices, while a negative correlation with FC was observed between the parietal operculum cortex and the cerebellum. Additionally, worse self-reported cognitive impairment and worse non-work daily functioning were associated with increased FC between regions involved in cognition and sensorimotor processing. These preliminary findings suggest that neural correlates for cognitive and daily functioning in glioma patients can be revealed using rs-fMRI. Resting-state network alterations may serve as a biomarker for patients’ cognition and functioning.

**Supplementary Information:**

The online version contains supplementary material available at 10.1007/s11682-021-00497-6.

## Introduction

Clinical outcomes for patients with diffuse gliomas, particularly lower grade gliomas, have significantly improved with aggressive chemoradiation (median survival increased from 7.8 to 13.3 years), but patients often suffer substantial changes to cognition and neurological functions (Buckner et al., 2016). Approximately 25% of lower grade glioma patients may report serious cognitive impairments including deficits in language, memory, attention, and executive function (Aaronson et al., 2011; Gehrke et al., 2013) stemming from several risk factors such as the effects of the tumor itself, surgery, and/or aggressive treatment (Barzilai et al., 2018; Gehrke et al., 2013; Gempt et al., 2017; Goldstein et al., 2004; Habets et al., 2014; Lang et al., 2017; Scheibel et al., 1996). Declines in cognitive and functional abilities for glioma survivors can have marked adverse effects on quality of life (Feuerstein et al., 2007; Mackworth et al., 1992). Thus, there is a growing emphasis on improving survivors’ quality of life by optimizing cognitive and functional abilities (Aaronson et al., 2011) and by using comprehensive neuropsychological test batteries to identify specific impairments to guide rehabilitation (Kyle R. Noll et al., 2018).

The presence of a tumor can also disrupt brain networks associated with specific cognitive functions. One powerful tool for studying brain networks is resting-state functional MRI (rs-fMRI), which is a non-invasive neuroimaging technique that allows patients to be scanned at rest, allowing for the evaluation of cognitively impaired patients for whom task performance can be challenging. This imaging method uses blood oxygen level-dependent (BOLD), low-frequency MRI signal oscillations in the brain to measure functional connectivity (FC) patterns of brain regions at rest (Biswal et al., 1995). Prior rs-fMRI studies in patients with brain tumors have shown that decreased FC is related to reduced overall survival (L. Liu et al., 2018), along with a number of impairments including visual deficits (Ying et al., 2020), motor deficits (Mallela et al., 2016; Otten et al., 2012), and cognitive deficits (Lang et al., 2017; Maesawa et al., 2015). However, only a few studies have evaluated FC alterations in patients with brain tumors following treatment (Kocher et al., 2020; Nenning et al., 2020), and their scope has been limited to patients with recurrent gliomas (Harris et al., 2014), patients with short-term follow-up (Vassal et al., 2017), and survivors of pediatric, not adult, brain tumors (Chen et al., 2016). One explanation on the limited number of longitudinal rs-fMRI studies is because fMRI is susceptible to artifacts that may arise around tumor resection cavities (Hua et al., 2017; Peck et al., 2009; Tomasi & Volkow, 2010). As a result, little is known on the relationships between FC and cognitive abilities in survivors of diffuse gliomas that may undergo significant treatment and disease-related deficits.

The goal of the current exploratory study was to determine potential associations between rs-fMRI FC and cognitive measures in survivors of diffuse gliomas using neuropsychological assessments and rs-fMRI analysis methods to account for tumor resection cavities. We hypothesized that cognitively impaired patients would have decreased FC in associated brain networks compared to non-impaired survivors. We also hypothesized that there would be relationships in FC with self-reported cognitive function, non-work daily functioning, and time since surgery.

## Methods

### Patient population

We recruited a total of 22 patients with the following inclusion criteria: 1) pathologically confirmed diffuse glioma (WHO II-IV); 2) completed all treatments and were radiographically stable with no disease progression for at least 6 months following surgery, radiation, and/or chemotherapy; and 3) were no longer on any active therapy. Consecutive patients seen in the Neuro-Oncology clinic at UCLA for routine follow-up of their glioma who met above criteria were recruited into the study, and all patients provided informed consent approved by the UCLA Institutional Review Board (IRB#11–001,876; Medical IRB Committee #3; University of California Los Angeles). All patients in this cross-sectional study underwent an rs-fMRI scan and a neuropsychological test battery. All analyses were done in compliance with the Health Insurance Portability and Accountability Act (HIPAA), and the UCLA IRB approved all aspects of the current study. The cohort included 15 males and 7 females, with a mean age of 43.8 years (range 22 to 70), as outlined in Table 1.

**Table 1:** Clinical data of patients

ID	Age	Sex	Tumor location	Tumor grade	IDH1/2 Status	Radiation	Chemotherapy	Antiepileptic treatment during MRI	Years since last treatment	Handed-ness
1	38	M	R FC	WHO II	Mutant	Y	Y	N	4.75	R
2	38	M	L FC	WHO III	Mutant	Y	Y	N	0.81	R
3	42	M	R PC	WHO III	Mutant	Y	Y	Y	4.41	R
4	39	M	L FC	WHO III	Mutant	Y	Y	N	3.81	R
5	50	F	L FC	WHO III	Unknown	Y	Y	Y	6.68	R
6	46	F	R FC	WHO IV	Mutant	Y	Y	Y	5.99	R
7	31	M	R FPC	WHO II	Mutant	Y	Y	Y	1.69	R
8	32	M	R TC	WHO III	Mutant	Y	Y	Y	3.84	R
9	41	M	R FC	WHO III	Mutant	Y	Y	Y	5.94	R
10	45	M	L FC	WHO III	Mutant	Y	Y	Y	2.77	R
11	62	M	R FC	WHO III	Mutant	Y	Y	Y	4.98	R
12	57	M	L FC	WHO IV	Mutant	Y	Y	Y	7.44	R
13	42	F	L OC	WHO IV	Wild Type	Y	Y	Y	6.40	R
14	61	F	R FC	WHO III	Mutant	Y	Y	Y	1.22	R
15	22	M	R FTC	WHO III	Mutant	Y	Y	Y	2.46	R
16	29	M	L TC	WHO II	Mutant	N	N	Y	4.49	R
17	70	M	R FC	WHO IV	Wild Type	Y	Y	Y	2.42	R
18	48	M	R PC	WHO IV	Mutant	Y	Y	Y	8.16	R
19	45	F	L PC	WHO III	Unknown	Y	Y	Y	12.37	R
20	46	M	L TC	WHO II	Mutant	Y	Y	Y	5.43	R
21	52	F	R FC	WHO II	Mutant	Y	Y	Y	0.70	R
22	28	F	L TC	WHO II	Mutant	Y	Y	N	2.35	R

*Y* Yes, *N* No, *M* Male, *F* Female, *R* Right, *L* Left, *FG* Frontal Cortex, *PC* Parietal Cortex, *TC* Temporal Cortex, *OC* Occipital Cortex, *FPC* Frontoparietal Cortex, *FTC* Frontotemporal Cortex, *WHO* World Health Organization, *IDH1/2* Isocitrate Dehydrogenase-1/2

### Cognitive and functional outcomes

Neurocognitive functioning was measured using the following neuropsychological test battery, with normed scores aggregated into domains: **Learning/Memory**—the Hopkins Verbal Learning Test – Revised (Brandt & Benedict, 2001); the Brief Visuospatial Memory Test – Revised (Benedict, 1997); **Attention/Processing Speed/Working Memory**—the Trail-Making Test Part A (Heaton et al., 2004; Reitan & Wolfson, 1985); the Wechsler Adult Intelligence Scale-IV Coding and Digit Span subtests; the Golden Stroop test (first two conditions) (Golden & Freshwater, 2002); Executive Function: verbal fluency/FAS test (Strauss et al., 2006); the Golden Stroop test (interference score); Trail Making Test Part B; **Language –** verbal fluency/animals; the Boston Naming Test (Kaplan et al., 2001); and **Visuospatial—**the Rey-Osterrieth Complex Figure—a visuospatial test, copy (Meyers & Meyers, 1995). Raw scores were transformed into standard Z-scores using published normative data. Cognitive Impairment was defined based on the International Cognition and Cancer Task Force (ICCTF) guidelines and accounting for the number of test scores in the battery: participants were categorized as impaired if they had two or more test scores ≤ -2 Z score, a more stringent criterion to limit the likelihood that we would falsely identify chance impairment (p < 0.05) (Ingraham & Aiken, 1996; Wefel et al., 2011).

Subjective cognitive functioning was assessed using the Functional Assessment of Cancer Therapy-Cognitive Function (FACT-Cog) version 3 (Wagner et al., 2009). The FACT-Cog yields four subscores derived from items using a 5-point Likert scale to rate impairment; we focused on the Perceived Cognitive Impairment (PCI) subscore, which is the generally preferred outcome from this instrument (https://www.facit.org/FACITOrg/Questionnaires; Wagner et al., 2009). The PCI score ranges from 0–72 with higher scores indicating better functioning. Daily functioning was measured using the Work Productivity and Activity Impairment (WPAI) instrument (Reilly et al., 1993). Since work status is often affected in many brain tumor patients, we specifically selected for the Ability measure of non-work functioning, which ranges from 1–10, with higher scores indicating more functional impairment.

### Resting-state fMRI acquisition and post-processing

All functional MR images were collected on a Siemens Prisma 3 T MR scanner (Siemens Healthcare, Erlangen, Germany) with a repetition time (TR) = 2000 ms; echo time (TE) = 28 ms; slice sickness of 4 mm with no interslice gap; field-of-view (FOV) of 220 mm with an acquisition matrix of 64 × 64 for an in-plane resolution of 3.4 mm, interleaved acquisition; and flip angle of 77°. Additionally, a 1 mm 3D isotropic MPRAGE sequence was acquired according to the standardized brain tumor imaging protocol (BTIP) (Ellingson et al., 2015).

**Fig. 1:**
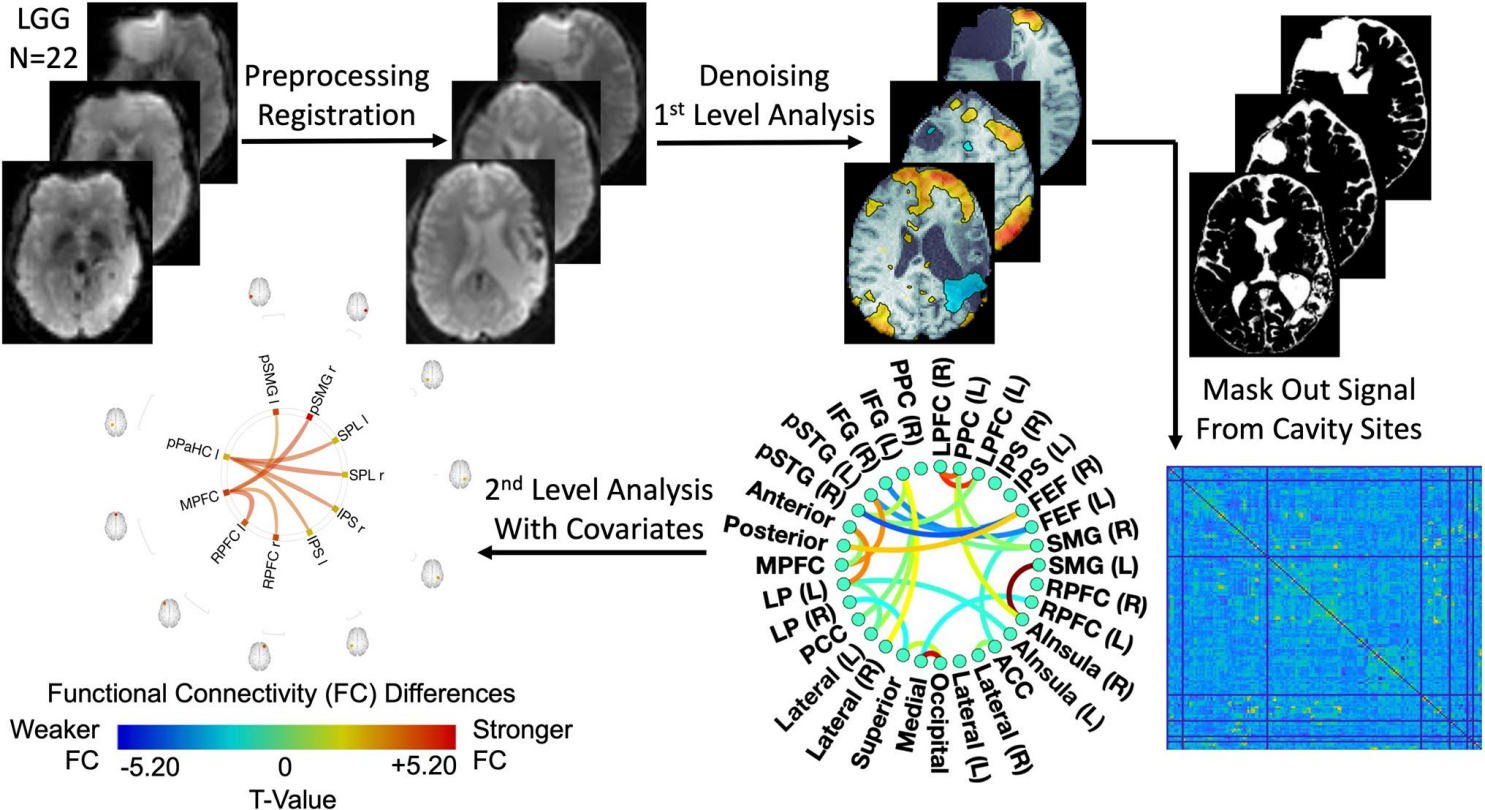
Statistical parameter mapping (SPM) pipeline for resting-state fMRI connectometry. All functional MR images underwent standard preprocessing steps and were registered to the MNI averaged T1 brain template. A band-pass filter of 0.008 Hz – infinity was used for denoising. After 1^st^ level analysis, individual tumor masks were created and applied to each patient’s connectome to remove signal from cavity sites. ROI-to-ROI functional connectivity (FC) analysis, which is based on general linear models (GLMs), was performed by associating FC with different types of clinical measurements. All analyses were controlled for patients’ age. Significance was set at p < 0.05 (two-sided) for the individual connections with a false discovery rate (FDR) < 0.05 based on the number of target regions

Resting-state FC analyses were performed using the CONN Toolbox (conn v.19.c https://www.nitrc.org/projects/conn) (Whitfield-Gabrieli & Nieto-Castanon, 2012), which implements functions from the Statistic Parametric Mapping (SPM12, http://www.fil.ion.ucl.ac.uk/spm/) toolbox (Ashburner & Friston, 2005). All functional MR images were pre-processed using the standard built-in preprocessing pipeline within CONN (Fig. 1), including functional realignment (motion correction, 12 degrees of freedom), unwarping, slice-timing correction, outlier detection (Artifacts Detection Tool via SPM package), registration of functional data to the structural volume, registration of the structural volume to the standardized space defined by the Montreal Neurological Institute (MNI) averaged T1 brain, and segmentation of structural volumes, which included skull stripping and processing of tissue types (GM, WM, and CSF). Spatial smoothing of the functional data was performed using an 8 mm full width at half maximum (FWHM) Gaussian kernel. Because rs-fMRI is interested in low-frequency oscillations (≤ 0.1 Hz), a band-pass filter of 0.008 – infinity Hz was applied for denoising data after regressing motion parameters and signal from the white matter (WM) and cerebrospinal fluid (CSF).

### Functional connectivity analysis

In order to evaluate patients’ resting-state network alterations, ROI-to-ROI (seed-to-seed) FC analysis was performed and associated with cognitive measures. All functional regions defined by the Harvard–Oxford atlas were initially selected as both seed and target ROIs for functional connectivity analyses. However, all patients in the present study previously underwent surgical resection, and surgical cavities are known to confound resting-state analyses (Tomasi & Volkow, 2010). As a result, individual tumor masks were drawn on anatomical MRI scans, and a novel algorithm was created to remove the Harvard–Oxford atlas ROIs present within the tumor mask from the FC analysis for each patient (see more in Supplemental Methods). We have also included classical networks that are commonly found in the resting-state literature, including the default mode, sensorimotor, visual, salience, dorsal attention, frontoparietal, language, and cerebellar networks. Those networks were well built and defined using CONN toolbox independent component analysis (ICA) of HCP (human connectome project) dataset (Whitfield-Gabrieli & Nieto-Castanon, 2012). After individual connectome and connectivity measures were generated, group analyses for associations between FC, the time since surgery, and cognitive measures for all 22 patients was conducted (Fig. 1). Due to the skewed distribution of the time since surgery, this data point was normalized using log-transformation before correlating it with FC. Age was used as a covariate and significance was set at p < 0.05 (two-tailed) for FCs with a false discovery rate (FDR) < 0.05 based on the number of target regions.

## Results

### Standard neuropsychological battery and self-assessment scores

Of the 22 patients in our study, 11 were categorized as cognitively non-impaired and 11 as cognitively impaired based on the International Cognition and Cancer Task Force (ICCTF) guidelines (Table 2). Statistical tests revealed no significant differences between cognitively non-impaired and cognitively impaired patients in sex (*p* = 0.361), race (*p* = 0.333), age (*p* = 0.194), education (*p* = 0.569), tumor location (*p* = 0.605) and grade (*p* = 0.605). All patients successfully completed the Work Productivity and Activity Impairment (WPAI) Test for non-work daily functioning and the Functional Assessment of Cancer Therapy-Cognitive Function (FACT-Cog) for self-reported cognitive impairment. The mean FACT-Cog PCI score was 43.2 (range 6 to 72), suggesting significant subjective cognitive impairment overall (Van Dyk et al., 2019). The mean WPAI Ability score was 2.6 (range 0 to 8), indicating relatively low functional impairment, though both had a wide range. Patient performances are summarized in Table 2.

**Table 2:** Patients’ performance on neuropsychological assessments

ID	Cognitively impaired	APW	EF	LANG	LM	FACT Cog PCI	WPAI act ability
1	N	0.03	0.70	0.55	0.78	67	0
2	N	0.45	0.40	0.00	0.90	42	5
3	N	-1.07	-0.67	-0.25	-0.10	N/A	N/A
4	Y	-1.75	-1.60	-1.15	-1.63	19	8
5	N	-0.57	-2.33	-1.15	-1.73	43	0
6	N	0.10	0.30	-0.05	-1.63	49	N/A
7	Y	-0.67	-0.57	-0.45	-2.30	6	6
8	Y	-0.91	-1.07	-1.25	-0.60	50	0
9	N	-0.73	-0.23	-0.25	0.18	N/A	3
10	N	-0.89	-0.30	-0.15	-0.53	59	3
11	Y	0.01	0.27	-0.05	-2.28	47	2
12	Y	-0.33	-2.03	-4.45	-2.48	38	8
13	Y	-0.67	-0.83	-1.15	-1.70	6	6
14	Y	-2.03	-1.67	-1.35	-3.00	35	0
15	N	0.27	1.10	-0.30	0.08	69	1
16	N	0.19	0.33	-0.40	0.90	72	0
17	Y	-1.24	-0.50	-1.75	-1.70	72	0
18	N	0.00	-0.43	-1.25	-1.60	47	2
19	N	-0.07	-0.47	0.35	-1.40	41	0
20	Y	-0.32	0.13	-2.75	-2.63	11	0
21	Y	-1.12	-1.17	-1.70	-1.98	41	6
22	Y	-1.28	-1.17	-3.20	-1.93	49	1
Summary (Mean ± SD) [Min, Max]	11/22 Impaired	-0.57 ± 0.67 [-2.03, 0.45]	-0.54 ± 0.90 [-2.33, 1.10]	-1.01 ± 1.22 [-4.45, 0.55]	-1.20 ± 1.19 [-3.00, 0.90]	43.2 ± 20.2 [6, 72]	2.6 ± 2.9 [0, 8]

*N* No, *Y* Yes, *SD* Standard Deviation, *N/A* Not Available, *APW* Attention, Processing speed, Working memory, *EF* Executive Function, *LANG* Language, *LM* Learning and Memory, *FACT Cog PCI* Functional Assessment for Cancer Therapy-Cognitive Function, *WPAI Act Ability* Work Productivity and Activity Impairment Test

### Functional connectivity in cognitively impaired and non-impaired survivors of glioma

**Fig. 2:**
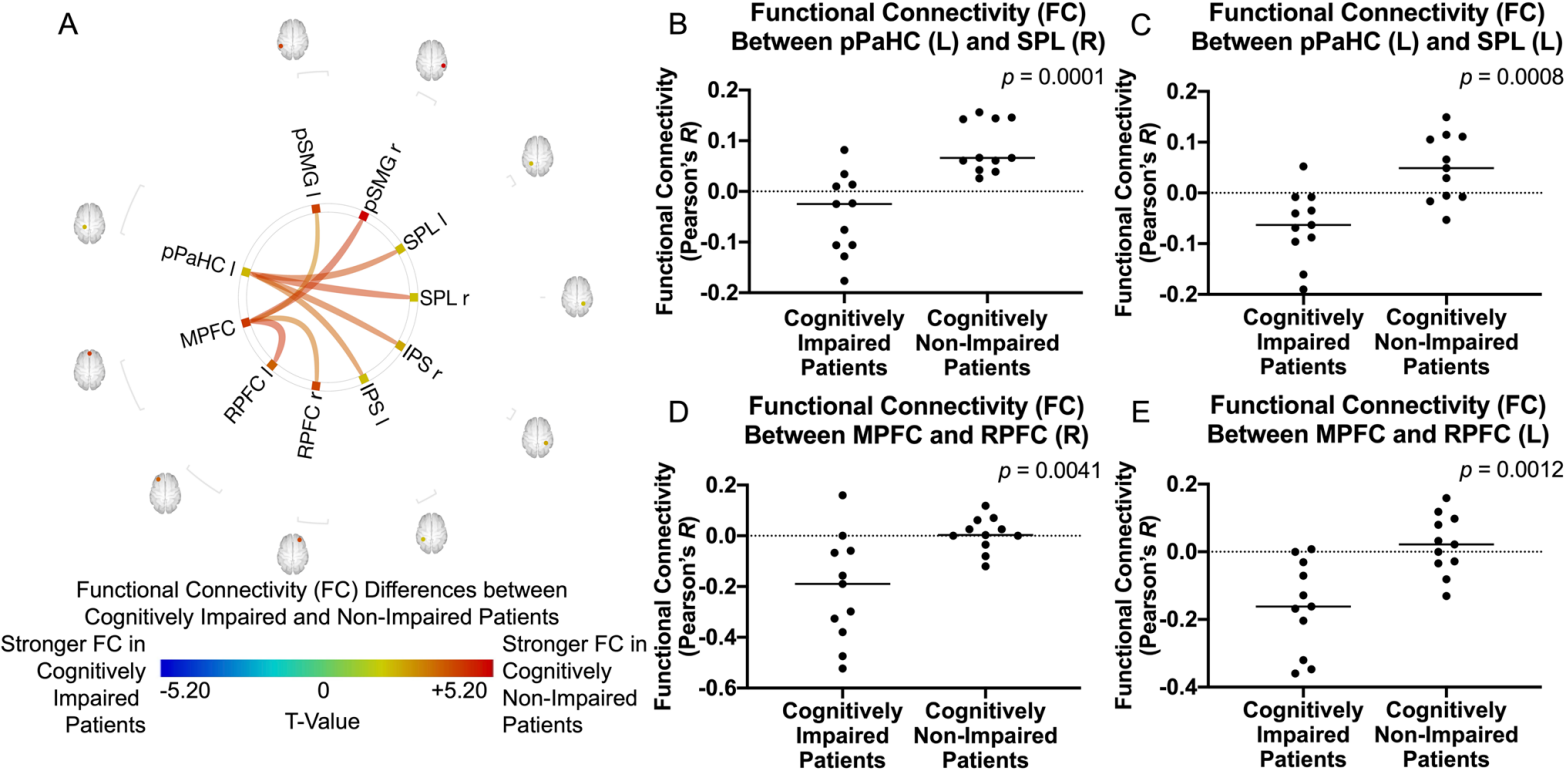
**A** Difference of ROI-to-ROI functional connectivity (FC) between cognitively impaired and non-impaired glioma patients after controlling for age. Colors denote value of the T-statistic, yellow–red represents stronger FC in cognitively non-impaired patients, cyan-blue denotes stronger FC in cognitively impaired patients. Position of ROIs displayed on mid-axial slices. SPL = Superior Parietal Lobule; pSMG = Supramarginal Gyrus, Posterior Division; pPaHC = Parahippocampal Gyrus, Posterior Division; MPFC = Medial Prefrontal Cortex; RPFC = Rostral Prefrontal Cortex; IPS = Intraparietal Sulcus; r = Right Hemisphere; l = Left Hemisphere. **B–E** Representative comparisons of FC between cognitively impaired and non-impaired patients, where **B** left pPaHC and right SPL (*p* = *0.0001*); **C** left pPaHC and left SPL (*p* = *0.0008*); **D** MPFC and right RPFC (*p* = *0.0041*); and **E** MPFC and left RPFC (*p* = *0.0012*)

Compared to the cognitively impaired patients, cognitively non-impaired patients had stronger FC of the medial prefrontal cortex (default mode network) with the bilateral posterior supramarginal gyri and the bilateral rostral prefrontal cortex (salience network). Moreover, stronger FC was observed between the left posterior parahippocampal gyrus, the bilateral intraparietal sulcus (dorsal attention network), and the bilateral superior parietal lobule in the cognitively non-impaired patients (Fig. 2).

### Functional connectivity associations with subjective cognition

**Fig. 3:**
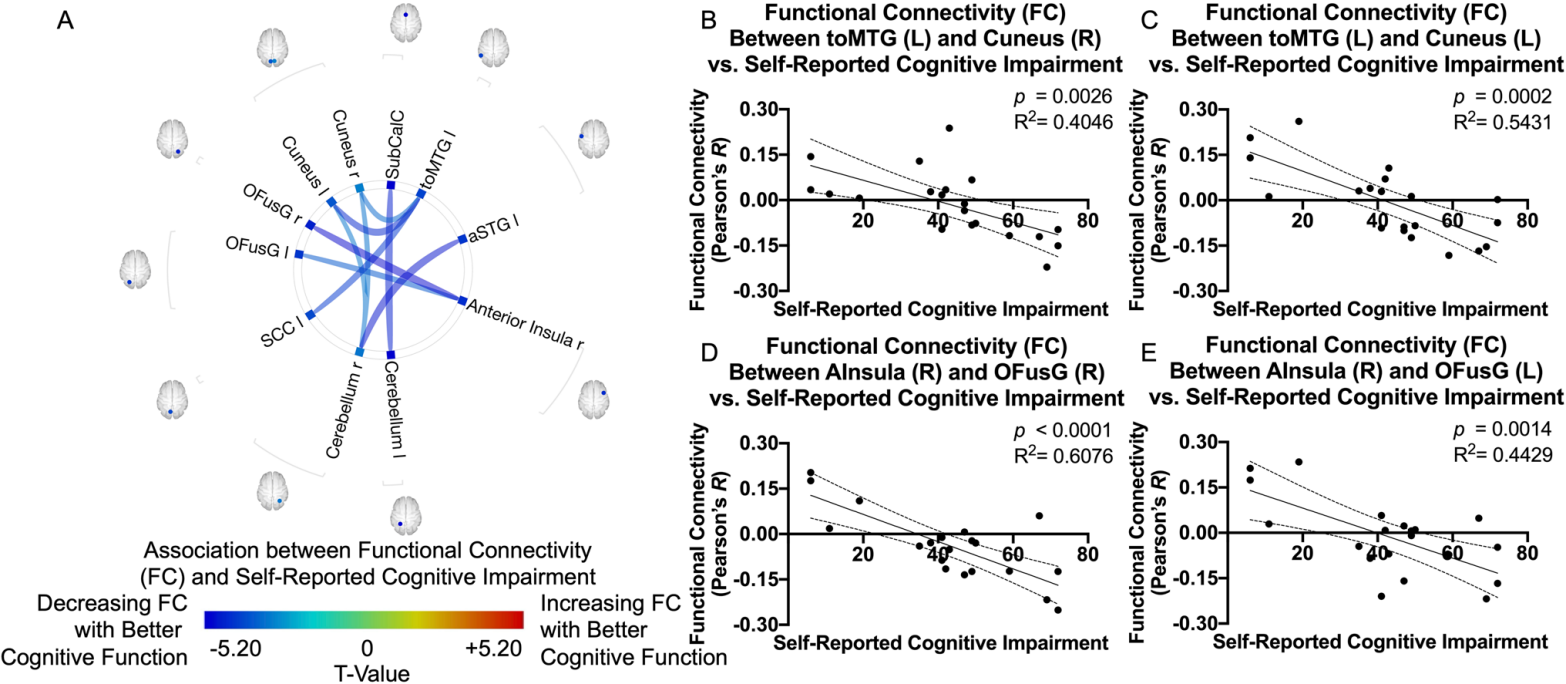
**A **ROI-to-ROI functional connectivity (FC) association with self-reported cognitive impairment for glioma patients after controlling for age. Colors denote value of the T-statistic, yellow–red represents positive association (increasing FC with better cognitive function), cyan-blue denotes negative association (decreasing FC with better cognitive function). Position of ROIs displayed on mid-axial slices. aSTG = Superior Temporal Gyrus, Anterior Division; toMTG = Middle Temporal Gyrus, Temporooccipital Part; SubCalC = Subcallosal Cortex; OFusG = Occipital Fusiform Gyrus; SCC = Supracalcarine; r = Right Hemisphere; l = Left Hemisphere. **B–E** Representative correlations observed between self-reported cognitive impairment and FC, where **B **left toMTG and right Cuneus (*R*^*2*^ = *0.4046, p* = *0.0026*); **C** left toMTG and left Cuneus (*R*^*2*^ = *0.5431, p* = *0.0002*); **D** right anterior insula and right OFusG (*R*^*2*^ = *0.6076, p* < *0.0001*); and **E** right anterior insula and left OFusG (*R*^*2*^ = *0.4429, p* = *0.0014*)

When examining the self-reported cognitive impairment, better subjective cognition was associated with decreasing FC between the right anterior insula and the bilateral occipital fusiform gyri. Additionally, the bilateral cuneus and the left supracalcarine cortex showed decreasing FC with the left temporo-occipital gyrus, while the cerebellum displayed decreasing FC with the bilateral cuneus, the subcallosal cortex, and the superior temporal cortex (Fig. 3).

### Functional connectivity associations with daily functioning

**Fig. 4:**
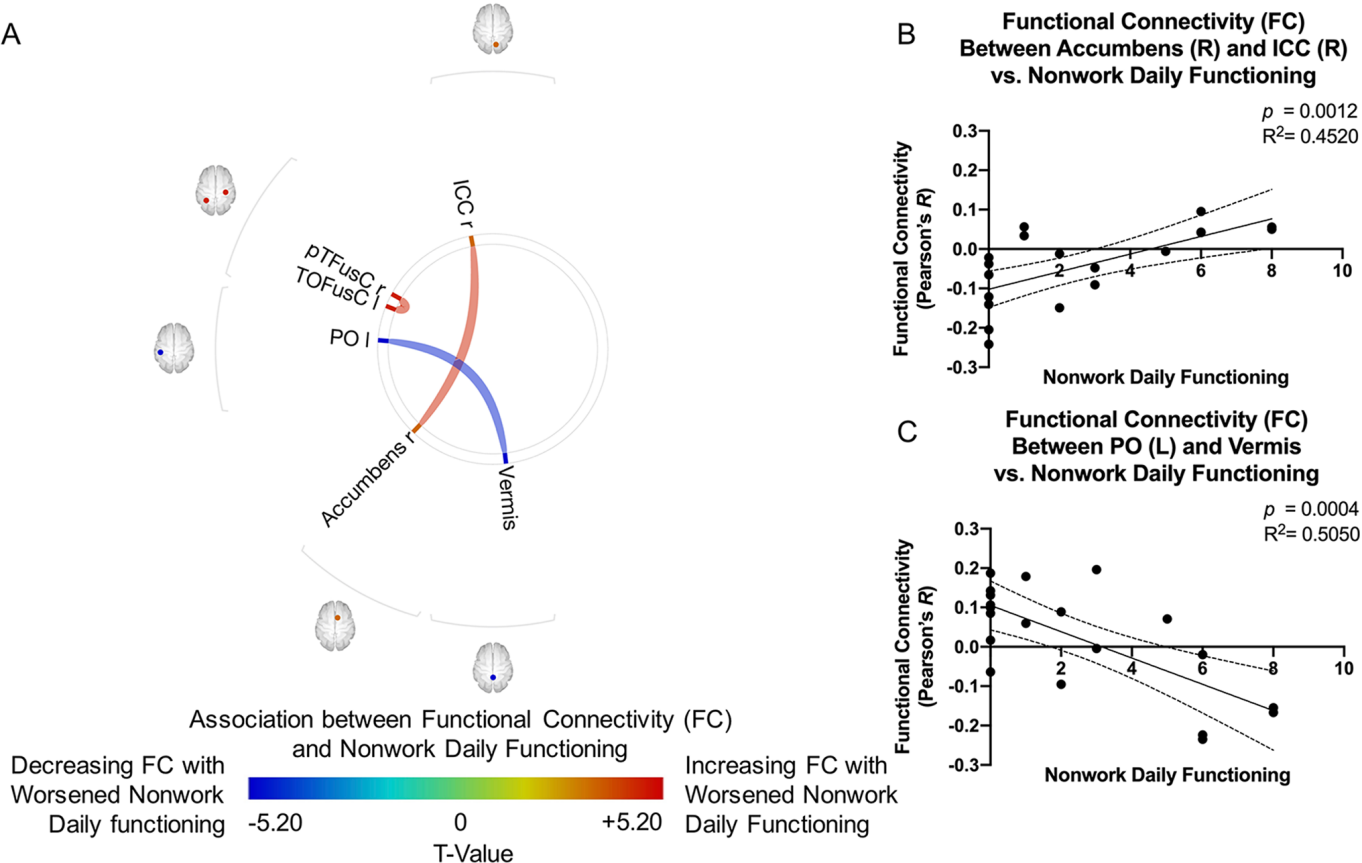
**A **ROI-to-ROI functional connectivity (FC) association with non-work daily functioning for patients with diffuse glioma after controlling for age. Colors denote value of the T-statistic, yellow–red represents positive association (increasing FC with worsened non-work daily functioning), cyan-blue denotes negative association (decreasing FC with worsened non-work daily functioning). Position of ROIs displayed on mid-axial slices. ICC = Intracalcarine Cortex; pTFuSC = Temporal Fusiform Cortex, Posterior Division; TOFusC = Temporal Occipital Fusiform Cortex; PO = Parietal Operculum Cortex; r = Right Hemisphere; l = Left Hemisphere. **B–C** Representative correlations observed between non-work daily functioning and FC, where **B** right Accumbens and right ICC (*R*^*2*^ = *0.4520, p* = *0.0012*); and **C** left PO and Vermis (*R*^*2*^ = *0.5050, p* = *0.0004*)

Worsened non-work daily functioning was associated with increasing FC between the right accumbens and the right posterior temporal fusiform cortex, as well as between the right intracalcarine cortex and the left temporal occipital fusiform cortex. Additionally, the cerebellum displayed decreasing FC with the left parietal operculum cortex with worsened non-work daily functioning (Fig. 4).

### Functional connectivity associations with other cognitive domains and functional assessments

Associations between FC and four cognitive domains: 1) Attention, Processing speed, Working memory (APW); 2) Executive Function (EF); 3) Language (LANG); 4) Learning and Memory (LM) were evaluated and reported in Suppl Figs. S1-S4.

### Relationships between functional connectivity and time since surgery

**Fig. 5:**
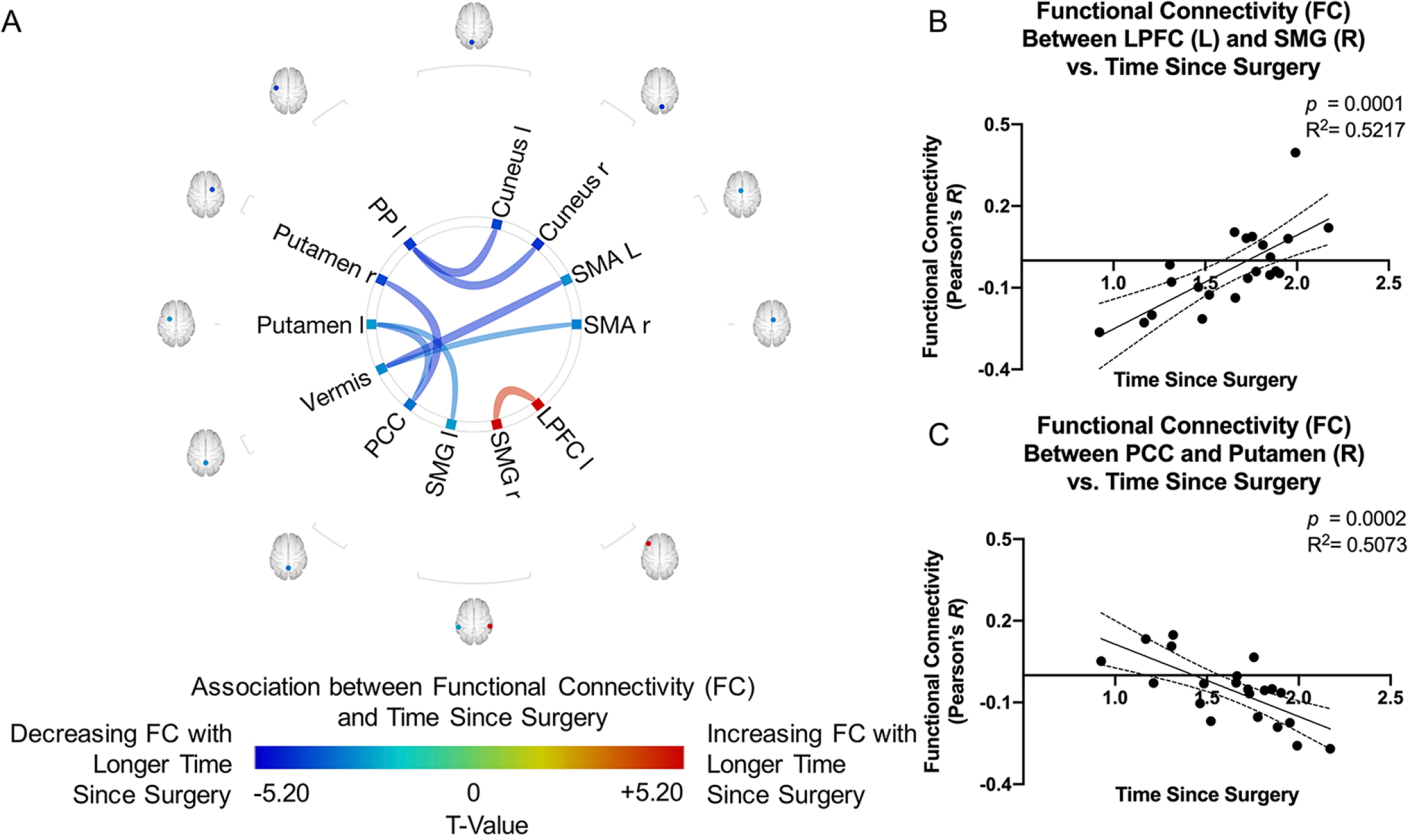
**A **ROI-to-ROI functional connectivity (FC) association with time since surgery for glioma patients after controlling for age. Colors denote value of the T-statistic, yellow–red represents positive association (increasing FC with longer time since surgery), cyan-blue denotes negative association (decreasing FC with longer time since surgery). Position of ROIs displayed on mid-axial slices. PP = Planum Polare; PCC = Posterior Cingulate Cortex; SMG = Supramarginal Gyrus; LPFC = Lateral Prefrontal Cortex; r = Right Hemisphere; l = Left Hemisphere. **B–E** Representative correlations observed between time since surgery and FC, where **B** left LPFC and right SMG (*R*^*2*^ = *0.5217, p* = *0.0001*); and **C **PCC and right Putamen (*R*^*2*^ = *0.5073, p* = *0.0002*)

Both negative and positive correlations were observed between FC and time since surgery (Fig. 5). A longer time since surgery was associated with increasing FC from the right supramarginal cortex (salience network) to the left lateral prefrontal cortex (fronto-parietal network). On the contrary, a longer time since surgery was associated with decreasing FC from the cerebellum to the bilateral SMA, from the posterior cingulate cortex (default mode network) to the bilateral putamen, and from the left planum polare to the bilateral cuneus. Also, the left supramarginal cortex (salience network) displayed decreasing FC to the left putamen associated with longer time since surgery.

## Discussion

Advanced multimodality treatments have led to the longer survival of patients with gliomas, but they also result in significant changes in cognition and quality of life (Feuerstein et al., 2007; Mackworth et al., 1992). This exploratory study examined associations between cognitive and functional measures with resting-state FC in diffuse glioma survivors, as defined by having stable disease for more than 6 months after completion of therapy and not on any active therapy during the time of evaluation. We observed widespread differences in FCs in cognitively impaired patients when compared to non-impaired patients. We also observed altered FCs associated with self-reported cognitive impairment, daily functioning, and time since surgery. These changes were observed in an array of brain regions implicated in memory, sensorimotor, and reward processing, such as the cingulate, cerebellum, cuneus, sensorimotor system, temporal, and frontal/prefrontal cortices. Combined with prior findings of altered resting-state networks and deterioration of cerebral neuroplasticity in glioma patients (Alcantara et al., 2019; Chen et al., 2016; Daniel et al., 2020; Fox & King, 2018; Lang et al., 2017; Noll et al., 2021; van Dokkum et al., 2019; van Nieuwenhuizen et al., 2018; Vassal et al., 2017), our results support the use of FC in studying cognitive and functional outcomes in this population.

Cognitively non-impaired patients showed stronger FC between salience network and default mode network nodes, as well as from the parahippocampal gyrus to the dorsal attention network. Improved cognitive function following surgical resection has already been previously observed (Barzilai et al., 2018; Vassal et al., 2017), and increased FC in the default mode network and salience network have been positively associated with better cognition in glioma patients (Chen et al., 2016; Fox & King, 2018; Maesawa et al., 2015; van Nieuwenhuizen et al., 2018). The parahippocampal gyrus is well-known for its role in learning and memory (Jayakar et al., 2015), and reduced FC from the parahippocampal gyrus has been associated with cognitive impairment in patients with Alzheimer’s Disease (Liu et al., 2016) and generalized anxiety disorder (Cui et al., 2016). Moreover, the dorsal attention network is implicated in goal-directed selective attention and focusing (Reineberg et al., 2018). A previous study on patients with tinnitus and hearing loss also observed increased FC between the dorsal attention network and parahippocampal gyrus. The authors suggested that this was a form of compensation to offload dorsal attention network activity to other brain regions (Schmidt et al., 2013). Interestingly, the brain is known to recruit atypical brain regions in the setting of tumors to compensate for affected regions (Cho et al., 2018; Li et al., 2019). While the present study did not compare changes in FC, the positive relationship between FC from the parahippocampal gyrus to the dorsal attention network in cognitively non-impaired patients may be one possible mechanism for preserved cognition in the non-impaired patient subgroup.

At the level of individual cognitive domains, we observed several associations across domains. There is a wealth of imaging literature implicating the role of the pre-frontal cortex tasks related to attention, working memory, and executive functioning in normal individuals (Kane & Engle, 2002; Lara & Wallis, 2015; Rossi et al., 2009). It is notable therefore that FC associations in the attention/processing speed/working memory and executive function domains predominantly did not involve frontal structures. Since the tumor region for a large proportion of our sample was in frontal regions, we inspected domain scores across participants/tumor location and could not discern a pattern based on performance in either domain; patients with a frontal tumor performed variably in both domains, so this does not likely fully explain this finding. Rather it raises further research questions about vulnerable networks and reorganization perhaps on a more granular level to support these cognitive abilities.

The correlation analysis with self-report measures of daily functioning, subjective cognitive impairment, and measures of mood revealed interesting relationships. The FC regions involved with poorer non-work daily functioning were positively associated between the accumbens and intracalcarine cortex, and negatively associated between the parietal operculum and cerebellum. The accumbens is a key region involved in the reward network (Wenzel et al., 2015). Previous analyses based on combined resting-state functional connectivity and meta-analytic connectivity modeling showed that spontaneous activity in accumbens of healthy participants predicts activity in regions implicated in reward circuitries, including sensorimotor cortex, cerebellum, and primary visual cortices (Cauda et al., 2011). Increased FC in the accumbens has been associated as a compensatory effect for defective reward network activity in patients with somatization disorder (Ou et al., 2019) and in patients with schizophrenia during cigarette cravings (Potvin et al., 2019). In addition, there were several associations between FC and measures of depression and anxiety symptoms, and in several overalapping networks with self-reported cognitive functioning and daily functioning. In the non-CNS cancer literature, self-reported cognitive functioning has been linked to mood and is actively investigated to understand quality of life for these patients (Hutchinson et al., 2012). In LGG survivorship, others have emphasized the need to better understand and address mood and quality of life symptoms. Our results may further suggest dynamic relationships across cognitive, functional, and mood outcomes, highlighting the need for more study. For instance, FC among regions involved in reward circuitry were observed in both analyses of self-reported functioning and mood, raising interesting research questions about shared contributing factors and substrate networks.

It is also worth noting the altered FC with the cerebellum. The cerebellum is broadly involved in the execution and network organization of many functions (Koziol et al., 2014). It has previously been associated with memory, verbal abilities, language, and visuospatial functions in patients with brain cancer (Cho et al., 2018; Zacharia & Eslinger, 2019). In the current study, the FC between the cerebellum and left fusiform was associated with performance in the language domain, echoing prior work implicating a link between these regions underlying orthographic processing (Booth et al., 2007). In addition, decreasing FC from the cerebellum to the bilateral SMA was correlated with time since surgery; decreasing FC from the cerebellum to the parietal operculum cortex was correlated with worsened non-work daily functioning; and decreasing FC from the cerebellum to the visual and auditory cortices was correlated with improved cognition. Decreased FC between the cerebellum and subcallosal cortex was also associated with improved subjective cognitive function. Similarly, we also found decreased FC between the cerebellum and right prefrontal cortex associated with high depression symptoms. The subcallosal cortex has been implicated in major depression and has been the target of deep brain stimulation treatment. Depression is a system-level disorder affecting integrated pathways linking select cortical, subcortical, and limbic sites and their related neurotransmitter and molecular mediators, antidepressant effects were associated with a marked reduction in local cerebral blood flow as well as changes in downstream limbic and cortical sites (Dunlop et al., 2017; Mayberg et al., 2005; Riva-Posse et al., 2014). These findings highlight the importance of studying cerebellar connections in glioma patients to uncover its role in neurobehavioral symptoms during survivorship.

### Limitations and future considerations

Some limitations of this pilot study should be highlighted. First, the dataset is comprised of a small population of glioma patients with a broad range of diagnoses, treatments, and time elapsed since their most recent treatment. Although this was a limitation, this heterogeneity also allowed for sufficient variation in cognitive performance across patients. Nevertheless, they are all considered "glioma survivors", as defined by having stable disease for more than 6 months after completion of therapy and not on any active therapy during the time of evaluation. Second, follow-up testing and longitudinal data are necessary to confirm observed association between FC and cognitive measures. Furthermore, group-level statistical analyses rely heavily on the precise construction of the FC connectome for each individual patient, which may be dependent on the location of the tumor resection cavity. In the present study, 14 of 22 patients had tumors involving the frontal lobes, and FC to ROIs located in the resection cavity were set to 0 to reflect the loss of the brain region and its associated function. However, this may have limited our ability to observe associations with frontal lobe regions in this study population by reducing overall FC magnitudes to this region. Nevertheless, we were still able to detect FC associations with existing brain regions, demonstrating the validity of this novel analysis method. Therefore, in future research we plan to continue evaluating FC for regions that were partially removed through surgery and examining a more variable distribution of tumor locations to further characterize the utility of this method. Third, due to the heterogeneity of the dataset with varying times post-treatment, a comparison of both cognitively-intact and cognitively-impaired datasets to an age-matched control dataset should be performed to further support the current findings. Additionally, analyses of connectivity patterns and their dependence on lesion location, including examining ipsilesional and contralesional networks and tumors in dominant or non-dominant hemispheres, should be examined in a larger cohort of patients. Lastly, a combination of multimodal images and correlation with other cognitive and motor measures should be employed to further characterize the cortical networks that are altered in diffuse glioma patients.

## Conclusion

Resting-state FC revealed several associations with cognitive and functional measures in a cross-sectional study of diffuse glioma survivors. The present findings suggest that FC alterations may be useful surrogates for cognition, daily functioning, and post-surgery recovery; however, future longitudinal studies with larger patient samples are needed to further probe the utility of these techniques.

## Supplementary Information

Below is the link to the electronic supplementary material.

Supplementary file1 (DOCX 656 KB)

Supplementary file2 (DOCX 28 KB)

## Data Availability

Data can be made available upon request through contacting the Corresponding Author.
