# Supplementary material for: Characterization of cognitive function in survivors of diffuse gliomas using resting-state functional MRI (rs-fMRI)
Source: Brain Imaging Behav. 2021 Aug 5;16(1):239–51. doi: 10.1007/s11682-021-00497-6 (PMC8825610; doi:10.1007/s11682-021-00497-6)
Supplement: Supplementary file 1 — Supplementary file1 (DOCX 656 KB) [file 11682_2021_497_MOESM1_ESM.docx]

**Fig. S1.** ROI-to-ROI functional connectivity (FC) association with better performance in cognitive domain of Attention, Processing speed, Working memory (APW). Colors denote value of the T-statistic, yellow-red represents positive association (increasing FC with better performance in cognitive domain of APW), cyan-blue denotes negative association (decreasing FC with better performance in cognitive domain of APW). Position of ROIs displayed on mid-axial slices. Full name of ROI abbreviations was listed in **Table S1**.


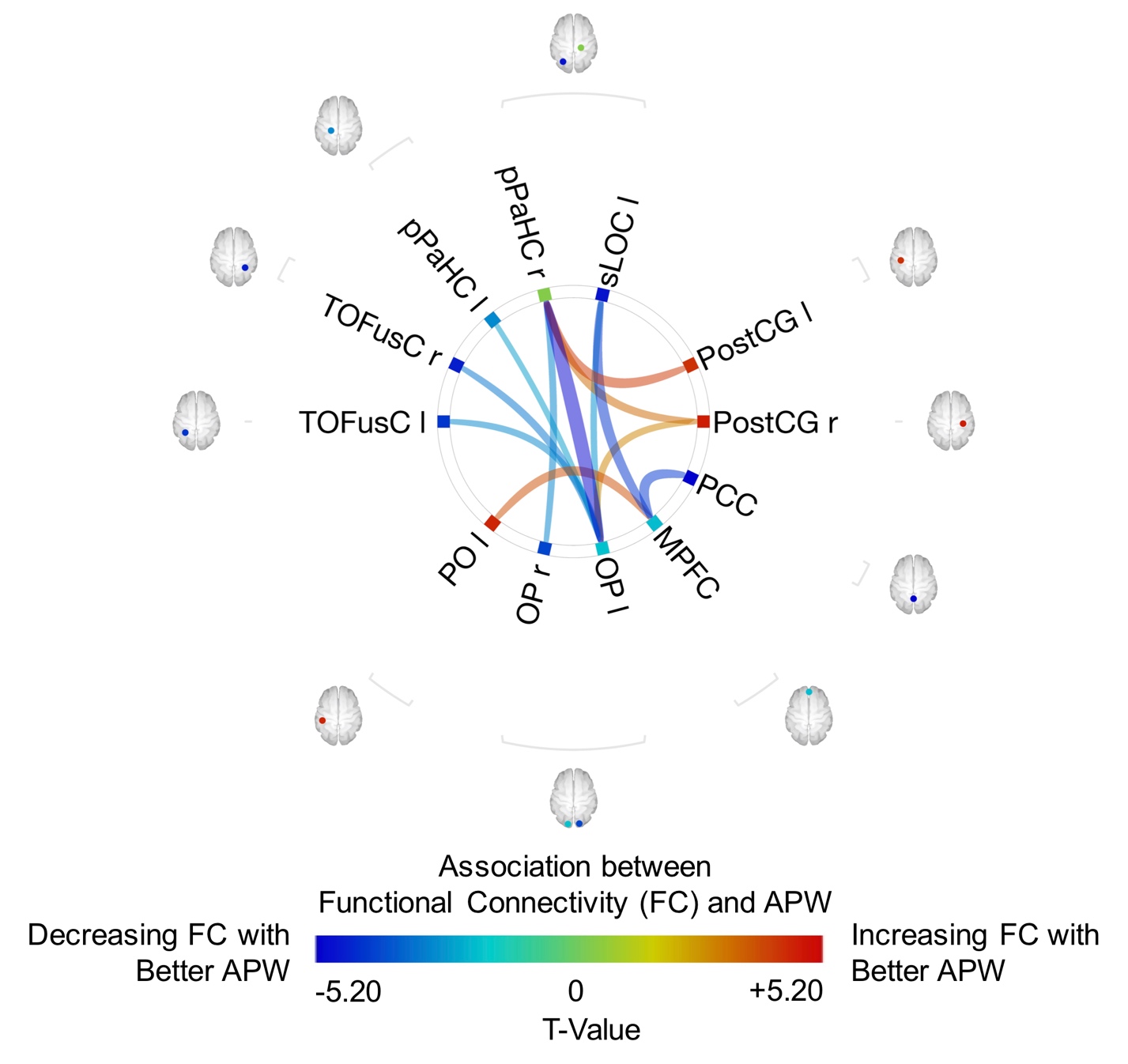


**Fig. S2.** ROI-to-ROI functional connectivity (FC) association with better performance in cognitive domain of Executive Function (EF). Colors denote value of the T-statistic, yellow-red represents positive association (increasing FC with better performance in cognitive domain of EF), cyan-blue denotes negative association (decreasing FC with better performance in cognitive domain of EF). Position of ROIs displayed on mid-axial slices. Full name of ROI abbreviations was listed in **Table S1**.


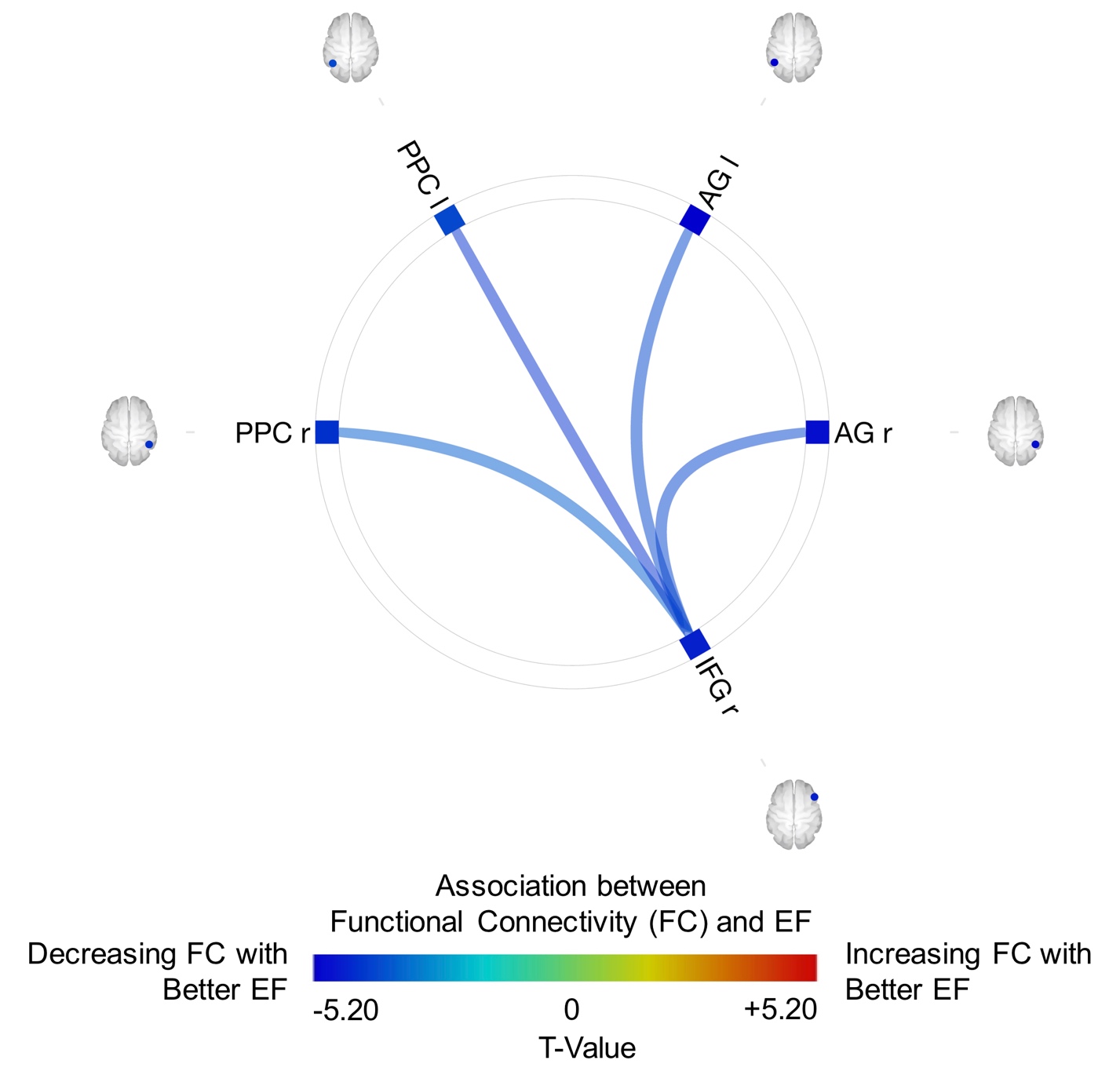


**Fig. S3.** ROI-to-ROI functional connectivity (FC) association with better performance in cognitive domain of Language (LANG). Colors denote value of the T-statistic, yellow-red represents positive association (increasing FC with better performance in cognitive domain of LANG), cyan-blue denotes negative association (decreasing FC with better performance in cognitive domain of LANG). Position of ROIs displayed on mid-axial slices. Full name of ROI abbreviations was listed in **Table S1**.


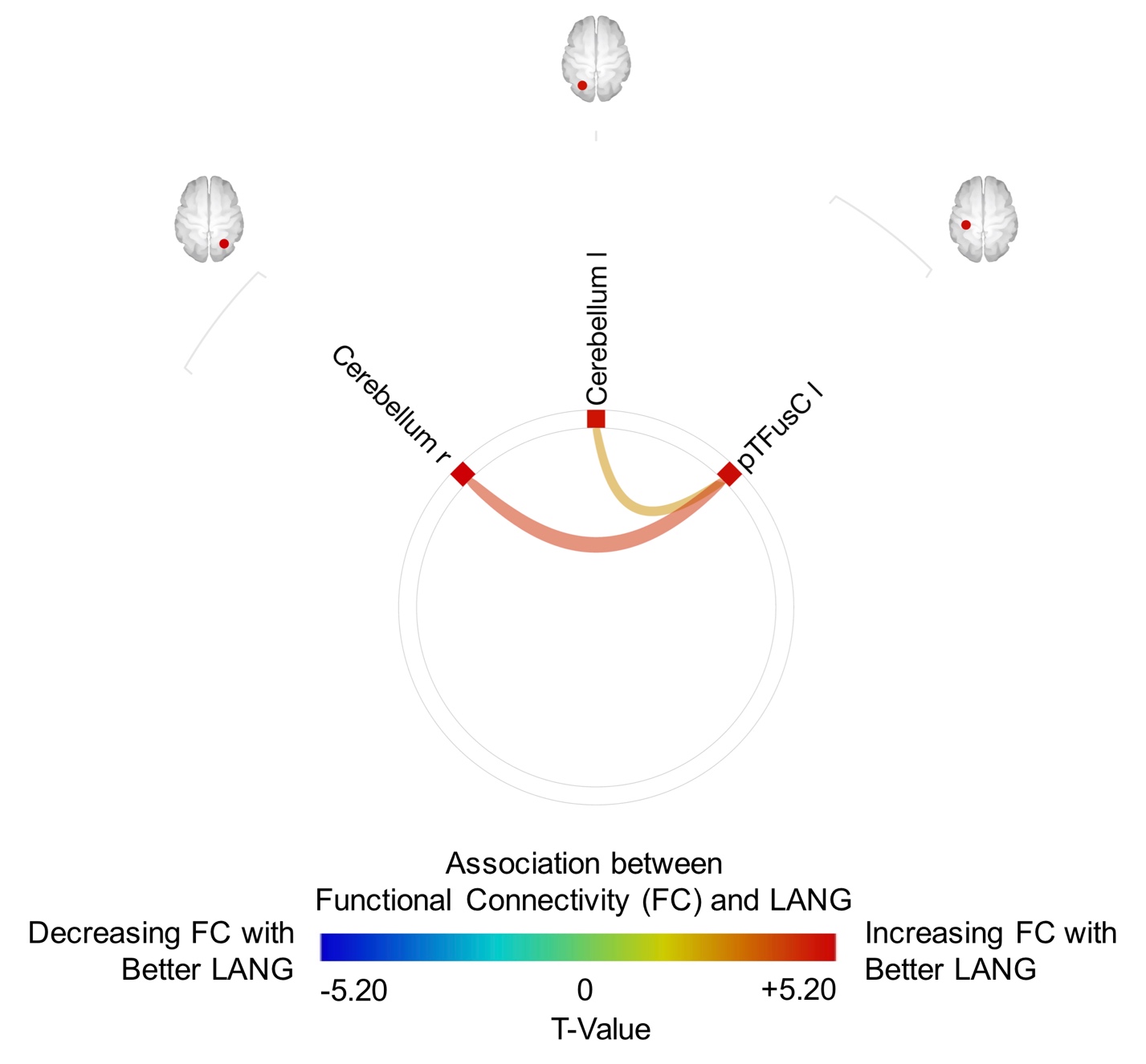


**Fig. S4.** ROI-to-ROI functional connectivity (FC) association with better performance in cognitive domain of Learning and Memory (LM). Colors denote value of the T-statistic, yellow-red represents positive association (increasing FC with better performance in cognitive domain of LM), cyan-blue denotes negative association (decreasing FC with better performance in cognitive domain of LM). Position of ROIs displayed on mid-axial slices. Full name of ROI abbreviations was listed in **Table S1**.


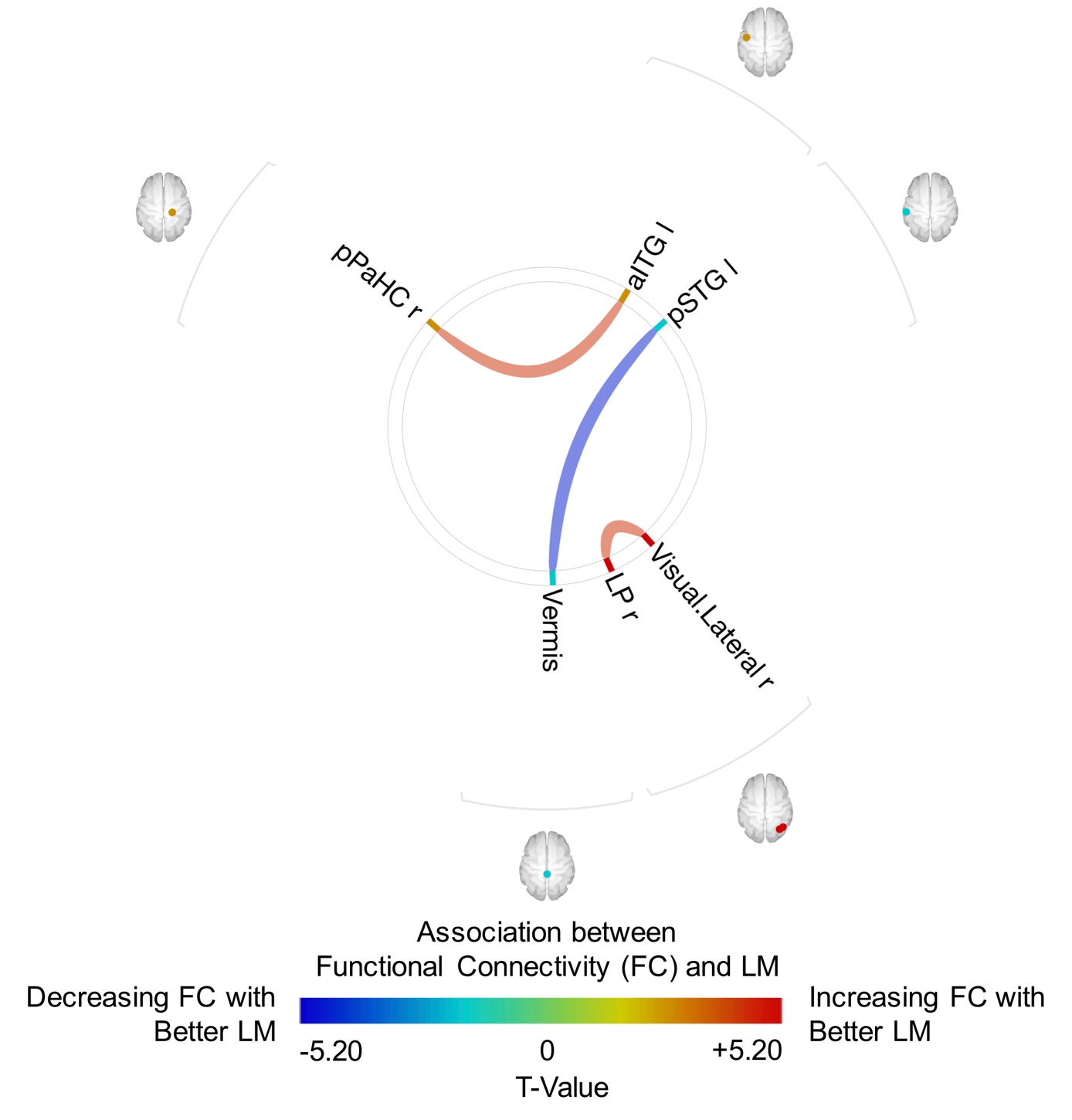


**Table S1.** Full name of region of interest (ROI) abbreviations for cortical regions.

| **Regions of Interest (ROIs)**  **Abbreviation** | **Full Name of Cortical Regions** |
| --- | --- |
| **IC** | Insular Cortex |
| **MidFG** | Middle Frontal Gyrus |
| **TP** | Temporal Pole |
| **pSTG** | Superior Temporal Gyrus, Posterior Division |
| **aITG** | Inferior Temporal Gyrus, Anterior Division |
| **pITG** | Inferior Temporal Gyrus, Posterior Division |
| **PostCG** | Postcentral Gyrus |
| **aSMG** | Supramarginal Gyrus, Anterior Division |
| **pSMG** | Supramarginal Gyrus, Posterior Division |
| **AG** | Angular Gyrus |
| **sLOC** | Lateral Occipital Cortex, Superior Division |
| **iLOC** | Lateral Occipital Cortex, inferior Division |
| **ICC** | Intracalcarine Cortex |
| **SubCalc** | Subcallosal Cortex |
| **PaCiG** | Paracingulate Gyrus |
| **AC** | Cingulate Gyrus, Anterior Division |
| **PC** | Cingulate Gyrus, Posterior Division |
| **aPaHC** | Parahippocampal Gyrus, Anterior Division |
| **pPaHC** | Parahippocampal Gyrus, Posterior Division |
| **LG** | Lingual Gyrus |
| **pTFusC** | Temporal Fusiform Cortex, Posterior Division |
| **TOFusC** | Temporal Occipital Fusiform Cortex |
| **OFusG** | Occipital Fusiform Gyrus |
| **CO** | Central Opercular Cortex |
| **PO** | Parietal Operculum Cortex |
| **OP** | Occipital Pole |
| **MPFC** | Medial Prefrontal Cortex, Default Mode Network |
| **LP** | Lateral Parietal, Default Mode Network |
| **PCC** | Posterior Cingulate Cortex, Default Mode Network |
| **ACC** | Anterior Cingulate Cortex, Salience Network |
| **RPFC** | Rostral Prefrontal Cortex, Salience Network |
| **SMG** | Supramarginal Gyrus, Salience Network |
| **PPC** | Posterior Parietal Cortex, FrontoParietal Network |
| **LPFC** | Lateral Prefrontal Cortex, FrontoParietal Network |
| **Visual.Medial** | Medial Visual Network |
| **Visual.Lateral** | Lateral Visual Network |
