# Supplementary material for: Characterization of cognitive function in survivors of diffuse gliomas using resting-state functional MRI (rs-fMRI)
Source: Brain Imaging Behav. 2021 Aug 5;16(1):239–51. doi: 10.1007/s11682-021-00497-6 (PMC8825610; doi:10.1007/s11682-021-00497-6)
Supplement: Supplementary file 2 — Supplementary file2 (DOCX 28 KB) [file 11682_2021_497_MOESM2_ESM.docx]

**SUPPLEMENTARY METHODS**

Functional Connectivity Analysis

In order to evaluate patients’ resting-state network alterations, ROI-to-ROI (seed-to-seed) functional connectivity (FC) analysis was performed and associated with cognitive measures. All functional regions defined by the Harvard-Oxford atlas were initially selected as both seed and target ROIs for functional connectivity analyses. However, all patients in the present study previously underwent surgical resection, and surgical cavities are known to confound resting-state analyses (Tomasi & Volkow, 2010). As a result, individual tumor masks were drawn on anatomical MRI scans, and a novel algorithm was created to remove the Harvard-Oxford atlas ROIs present within the tumor mask from the FC analysis for each patient.

*Creating Patient-Specific Tumor Mask*

The CONN toolbox constructs the FC networks of gray matter (GM) regions in the brain based on temporal co-variations of the blood oxygenation level-dependent (BOLD) signals (Whitfield-Gabrieli & Nieto-Castanon, 2012). The standard built-in preprocessing pipeline within the CONN toolbox construed the surgical cavities of individual patients to be comprised of either white matter (WM) or cerebrospinal fluid (CSF). Therefore, we visually reviewed the registered WM mask and CSF mask for each patient following the skull stripping and segmentation to confirm that the WM and CSF masks indeed contained the surgical cavities. After that confirmation, we merged the WM and CSF masks to create patient-specific tumor mask.

*Creating Individual Connectome and Connectivity Measures*

After utilizing the CONN toolbox’s standard built-in preprocessing pipeline, we overlapped the patient-specific tumor mask with each functional region as defined by the CONN toolbox’s default atlas. Where we found an overlapping cluster for a specific functional region, we further calculated the volume for both the overlapping cluster and the functional region. We assumed that no BOLD signal can be detected from a specific functional region if the volume of the overlapping cluster exceeded 95% of the volume of that specific functional region, and thus we set the FC for that region at 0. Once all functional regions were overlapped with patient-specific tumor masks, an updated adjacency matrix containing the inter-regional FC was used to construct the individual connectomes.

After individual connectome and connectivity measures were generated, group analyses for associations between FC, the time since surgery, and cognitive measures for all 22 patients was conducted (**Fig. 1**). Due to the skewed distribution of the time since surgery, this data point was normalized using log-transformation before correlating it with FC. Age was used as a covariate and significance was set at p < 0.05 (two-tailed) for FCs with a false discovery rate (FDR) < 0.05 based on the number of target regions.

**REFERENCES**

Tomasi, D., & Volkow, N. D. (2010). Functional connectivity density mapping. *Proc Natl Acad Sci U S A, 107*(21), 9885-9890. doi:10.1073/pnas.1001414107

Whitfield-Gabrieli, S., & Nieto-Castanon, A. (2012). Conn: a functional connectivity toolbox for correlated and anticorrelated brain networks. *Brain Connect, 2*(3), 125-141. doi:10.1089/brain.2012.0073
